# Rudiments of Animal Physiology, for Use in Schools, and for Private Instruction

**Published:** 1840-04

**Authors:** 


Art. VI.—*Rudiments of Animal Physiology, for use in Schools, and for Private Instruction.* By Dr. G. Hamilton, Falkirk. (*In Chambers's Educational Course.*)—*Edinburgh*, 1839. 18mo, pp. 104.

It always gives us pleasure to notice any work in which the elementary principles of physiology are adapted to popular comprehension; since we feel assured that the general diffusion of sound knowledge on this subject is intimately connected with the progress of mankind in *all* those objects which the true philanthropist regards with the deepest interest,—the physical, moral, and intellectual improvement of his race. During the whole course of our labours, we can truly say, we have not met with a stronger claim upon our warm approbation than that which this unpretending little work possesses. As one of a series which is already producing a great improvement in popular education, especially in Scotland, we were predisposed in its favour; and a careful examination of the treatise itself has satisfied us that it is *by far the best* of its class, in point of matter and illustration, whilst it is at the same time the cheapest. It possesses a great advantage over all others that we have seen, in being written by one who has himself been concerned in giving elementary instruction on the subject. Dr. Hamilton mentions in his preface that, under his superintendence, the master of the Falkirk parochial school has for the last four years made physiology a part of the regular course of instruction; with the two good effects of interesting his pupils and communicating to them knowledge of practical value. This circumstance has led him to append to each section of his work a list of books which may be advantageously referred to by the teacher, and also concise instructions for the preparation and exhibition of the parts described. The woodcut illustrations are numerous and well executed; those illustrative of the circulating system are coloured. These illustrations are principally taken from the "Elemens de Zoologie," an elementary work by one of the first naturalists now alive, of which we formerly spoke with high approbation. In this country, men of scientific reputation think it beneath them to write elementary books; and hence these are generally the production of men of superficial acquirements, who introduce into them much that the student has to unlearn if he advances further. We have looked through this little book with much attention, for the sake of being able to give a positive opinion on its scientific merits; and we have much pleasure in stating that it appears to us to give *sound* and *lucid* views on almost every subject discussed. The only point of consequence in which we think that the work might be improved, is in the account given of the respiratory process, and of the dependence of animal heat upon it. Dr. H. does not seem to be aware how universally Crawford's theory is *now* abandoned by the best-informed physiologists, and how readily the phenomena of animal heat are explicable upon the doctrine now generally received. As he quotes Dr. Alison in support of his views, we would refer him to p. 251 of the *last* edition of Dr. A.'s "Outlines;" and also to chap. xii. of Dr. W. B. Carpenter's "Principles of Physiology." The page in question might be easily cancelled and replaced by another; and we should then regard the work as unexceptionable. We do not think that our professional brethren can do a better service to their younger friends, and to schools with whose conductors they have any influence, than by urging its employment amongst them. We hope that this is not the only time that Dr. Hamilton will present himself to the public.

The true bearing of works like the present, and their signal importance to the public, are so admirably stated in the *editor's preface*, that we cannot resist the pleasure of making an extract from it, and with this we shall conclude our notice:

"While it has been justly objected to popular medical guides and dictionaries that, read under the influence of imperfect knowledge, they tend rather to mislead than to instruct, and probably induce more diseases than they cure, a candid mind must regard in a very different light the various works which have been published of late years for the purpose of conveying a popular knowledge of animal physiology. The sole and certain result of these must be, by giving a familiar knowledge of the human organization and its laws, to put individuals into the best possible condition for *avoiding* diseases—a very different thing, indeed, from the attempt to *cure* them. The utility of this knowledge to the non-medical community is now beginning to be generally felt, though still some perhaps require to be convinced of it. To such persons it might be pointed out that, though almost all, from the communications made to them in childhood, or from their own sensations and experience, are enabled to observe some of the more obvious laws of organization—as, for a familiar instance, those respecting simple overloading of the stomach,—there are others of those laws which most persons, for want of knowledge, are constantly breaking, to the great injury of their health. The general prevalence, amongst men of business, of an overtasking of the brain; amongst ladies, of a neglect of out-of-doors exercise; the almost universal indulgence in stimuli of various kinds; and the tight-lacing of young females; are but a scantling of the errors which we everywhere see around us, as the result of a want of knowledge of the structure and functions of our physical frames. It is only, indeed, where the infraction of any organic law is followed very immediately by its appropriate penalty, as simple overeating is by indigestion, that ordinary knowledge observes and records the fact. In the far more numerous, and generally much more important class of cases, where the effect is not to be readily traced to its cause, popular knowledge is completely at fault: nothing can there be of avail but a knowledge to some extent of the human organization and its laws. It may be true that the knowledge itself will not be sufficient to produce, in all, a proper attention to the rules of health; yet it is pretty clear that the knowledge *may* have such an effect, while without it nothing of the kind can be hoped for. It might also be expected that, were a knowledge of our internal organization thoroughly familiarized and made present to every mind, public opinion would become engaged in causing individuals of a negligent disposition to observe the laws of health. It would be thought wrong for a man having a family depending on himself to expose his life to hazard by daily-endured mental exhaustion; and a young lady entering a room with a waist reduced to half its proper circumference, would be shrunk from as a kind of monster. This knowledge would operate not only in a direct way upon individuals, but through one individual upon another."

